# Efficient Deamination of 5-Methylcytidine and 5-Substituted Cytidine Residues in DNA by Human APOBEC3A Cytidine Deaminase

**DOI:** 10.1371/journal.pone.0063461

**Published:** 2013-06-20

**Authors:** Rodolphe Suspène, Marie-Ming Aynaud, Jean-Pierre Vartanian, Simon Wain-Hobson

**Affiliations:** Molecular Retrovirology Unit, Institut Pasteur, Paris, France; Institut Pasteur Korea, Republic of Korea

## Abstract

Deamination of 5-methylcytidine (5MeC) in DNA results in a G:T mismatch unlike cytidine (C) deamination which gives rise to a G:U pair. Deamination of C was generally considered to arise spontaneously. It is now clear that human APOBEC3A (A3A), a polynucleotide cytidine deaminase (PCD) with specificity for single stranded DNA, can extensively deaminate human nuclear DNA. It is shown here that A3A among all human PCDs can deaminate 5-methylcytidine in a variety of single stranded DNA substrates both *in vitro* and in transfected cells almost as efficiently as cytidine itself. This ability of A3A to accommodate 5-methyl moiety extends to other small and physiologically relevant substituted cytidine bases such as 5-hydroxy and 5-bromocytidine. As 5MeCpG deamination hotspots characterize many genes associated with cancer it is plausible that A3A is a major player in the onset of cancer.

## Introduction

The human APOBEC3 (A3) locus encodes a seven gene cluster of PCDs [1]. While several clearly function as restriction factors for retroviruses and DNA viruses, their roles in the absence of infection are largely undefined [2]–[17]. While transfected plasmid DNA can be hyperedited by A3A, A3C and A3H, A3A is by far the most efficient of the three enzymes [15], [18]. Furthermore, human mitochondrial DNA (mtDNA) in the cytoplasm is susceptible to cytidine deamination, probably by one or more A3 PCDs [19]. Singularly, only A3A could deaminate human nuclear DNA (nuDNA). As observed levels of mt and nuDNA editing were dependent on uracil DNA glycosylase, it was suggested that A3 editing of human DNA was part of a novel DNA catabolic pathway [19]. 5-methylcytidine is the most common DNA modification and cytidine methylation is a major epigenetic mechanism of gene regulation and development. 5MeCpG mutation hotspots within cancer associated genes have long been described, the CpG motif being dictated by the host methylase while deamination is considered to be spontaneous [20]–[23]. Some reports suggest that 5MeC deamination is kinetically favoured over C deamination [24], [25], while others have suggested that DNA repair of the T:G base pair with respect to a U:G pair contributes to the so called mutation hotspots [26]–[28].

5-methycytidine is not the only naturally occurring cytidine derivative in DNA. Activated neutrophils and eosinophils in particular generate high levels of hydrogen peroxide, HOCl and HOBr. Cytidine can be oxidized by ^•^OH, ^•^OCl, and ^•^OBr free radicals to yield 5-hydroxy, 5-chloro and 5-bromocytidine derivatives [29]–[31]. Ultimately these bases have to be catabolyzed. Generally the cytidine heterocycle is oxidized first by deamination to uracil, then barbituric acid derivatives and finally urea and malonic acid. As we have argued that A3A plays a physiological role in the catabolism of nuDNA [19], the question arose as to whether A3A could also oxidize 5-substituted cytidine bases in DNA. It is shown here that A3A is the only human PCD that edits 5MeC efficiently as well as 5-hydroxy and 5-bromo-derivatives.

## Materials and Methods

### Cell Culture and Transfections

Japanese quail embryonic fibroblast QT6 cells (ATCC CRL 1708) were maintained in Ham’s medium supplemented with 100 units/ml penicillin, 2 mM glutamine, 5% tryptose phosphate, 1% chicken serum and 10% fetal calf serum. 6×10^5^ QT6 cells in 6 well-plates were transfected with functional A3 expression plasmids (2 µg), the A3A_C101S_ catalytic mutant or pv (empty vector) and one day later, transfected with heat denatured T5MeCGA DNA (200 ng) using JetPrime (Polyplus Transfection, USA), total DNA was recovered at 48 hours post initial transfection and extracted using the MasterPure Complete DNA and RNA purification kit (Epicentre).

### PCR and 3DPCR

dCTP, 5Me-dCTP, 5BrdCTP, 5HOdCTP, 5CH_2_OHdCTP and 5IdCTP were from Trilink (USA). A 679 bp fragment corresponding to part of the HIV-1 LAI *env* gene was amplified using total substitution of dCTP by 5Me-dCTP (Trilink, USA) using the primer pair MC1, 5′TTGATGATCTGTAGTGCTACAGAA and MC2, 5′GCCTAATTCCATGTGTACATTGTA. The first reaction involved standard amplification, reaction parameters were: 95°C for 5 min., followed by 35 cycles (each consisting of 95°C for 1 min., 53°C for 30 sec., and 72°C for 2 min.), and finally 20 min. at 72°C. The second PCR reaction involved standard amplification, reaction parameters were: 95°C for 5 min., followed by 30 cycles (each consisting of 95°C for 45 sec., 54°C for 45 sec., and 72°C for 90 sec.), and finally 20 min. at 72°C, primers were MC3, 5′TGTACCCACAGACCCCAACCCACAA and MC4, 5′TTCCATTGAACGTCTTATTATTACA. Differential amplification occurred in the third round by using an Eppendorf gradient Mastercycler S [16], [32], [33]. The reaction parameters were 78–90°C for 5 min., followed by 35 cycles (each consisting of 78–90°C for 45 sec., 56°C for 45 sec., and 72°C for 90 sec.), and finally 20 min. at 72°C, primers were MC5, 5′ATCAAAGCCTAAAGCCATGTGTAA and MC6, 5′CAATAATGTATGGGAATTGGCTCAA. 3DPCR products were cloned into the pCR2.1 TOPO cloning vector (Invitrogen) and sequenced (GATC).

Ten pmol of 5MeC containing oligonucleotides (Sigma) CER, 5′AGGAGTGGATGGGATTAGGGTGCGAATCMeCGAATTCGAATGMeCGAATACGAATTMeCGAATCCGAATAMeCGAATTGGAGGGTGTGAGTGTGGA were incubated with 10 ng of purified A3A-myc-His [18] or 10 ng of baculovirus produced A3G [1], [34] for 3 hrs at 37°C in 40 µl of 50 mM Tris.HCl pH7.4, 10 mM EDTA. Standard DNA was recovered by 30 rounds of standard PCR using the primers pairs 5′AGGAGTGGATGGGATTAGGG and 5′TCCACACTCACACCCTCCAA. Amplification, reaction parameters were: 95°C for 5 min., followed by 30 cycles (each consisting of 95°C for 30 sec., 57°C for 30 sec., and 72°C for 1 min.), and finally 20 min. at 72°C. The reaction parameters for 3DPCR were 75–84°C for 5 min., followed by 35 cycles (each consisting of 75–84°C for 30 sec., 57°C for 30 sec., and 72°C for 1 min.), and finally 20 min. at 72°C. 3DPCR products were cloned into the pCR2.1 TOPO cloning vector (Invitrogen) and sequenced (GATC).

The oligodeoxynucleotides TP53 exon 8, 5′AGGAGTGGATGGGATTAGGG-(TTGAGGTGMeCGTGTTTGTGCCTGTCCTGGGAGAGACMeCGGCGCACA)-TTGGAGGGTGTGAGTGTGGA were incubated with 10 ng of purified A3A-myc-His [18] in the same conditions of CER. Amplification reaction parameters were: 95°C for 5 min., followed by 30 cycles (each consisting of 95°C for 30 sec., 57°C for 30 sec., and 72°C for 1 min.), and finally 20 min. at 72°C. Primers were identical to CER amplification. PCR products were cloned into the pCR2.1 TOPO cloning vector (Invitrogen) and sequenced (GATC).

## Results and Discussion

**Figure 1:**
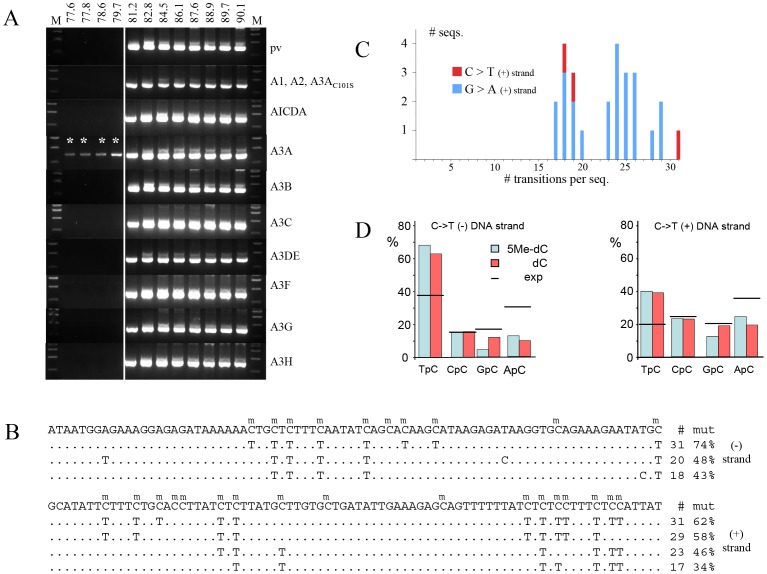
A3A deamination of 5-methylcytidine in ssDNA. A) Agarose gels of 3DPCR products derived from PCD transfected QT6 cells by denatured totally 5MeC substituted HIV *env* DNA. The temperatures refer to the differing PCR Tds used. The minimal Td for unedited DNA is 81.2°C. pv = plasmid expression vector. DNA (310 bp) recovered at Td<81.2°C, with an astericks, were cloned and sequenced. This experiment was performed in triplicate. B) A selection of A3A deaminated 5MeC substituted HIV DNA sequences (only 80 bp of 310 bp were presented); only differences are shown. To the left are the number and % of 5MeC bases deaminated per sequence. C) Distribution of edited sequences recovered from the 79.7°C reaction. D) Dinucleotide preference for A3A deamination of 5MeC (blue) and C (red). There was no 3′ effect. The expected (exp) values based on the dinucleotide composition of the plus and minus strands are given by horizontal lines.

A totally 5MeC substituted 685 bp fragment of HIV DNA was made by PCR and co-transfected into QT6 cells along with the cytidine deaminase expression plasmid. QT6 cells do not produce an endogenous cytidine-editing background because the avian lineage does not encode A1 or A3 othologs [16], [32], [35], [36]. After 24 hours, total DNA was recovered and amplification of an internal 485 bp fragment was made by standard PCR. Subsequently a nested 310 bp fragment was recovered by 3DPCR [33], a technique that allows recovery of AT rich DNA by exploiting its lower denaturation temperature (Td). As can be seen ∼81.2°C represents the lowest Td of input DNA (Figure 1A). Of the eight functional human PCDs only for A3A were 3DPCR products recovered at lower temperatures, down to 77.6°C. When sequenced these products proved to be hypermutated (mean 42%, range 30–66% methylcytidines edited, Figures 1B and 1C). There was a strong preference for editing in 5′TpC dinucleotides on both strands, a trait of A3A, which was not altered by the 5-methyl group (Figure 1D) [19]. The negative findings for all the other active PCDs show that A3A deamination of traces of input normal DNA used for making the totally substituted 5MeC containing DNA was not responsible for the 3DPCR signals (Figure 1A).

**Figure 2:**
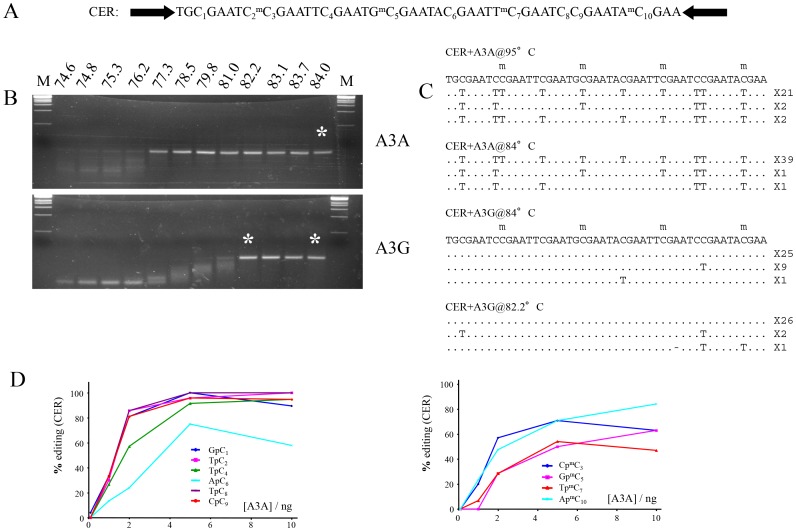
Similar A3A editing of 5MeC and C *in vitro.* A) The CER target sequence is nested between two PCR primer targets (black arrows). Every 5MeC site has matched non-methylated equivalent to allow comparisons. B) 3DPCR recovery of A3A edited CER DNA. A3A-myc-His was purified from HEK293T cells as described [18]. The asterisks denote the PCR products cloned and sequenced. C) A selection of deaminated CER sequences with the number of mutant sequences shown to the right. D) Site specific cytidine and 5-methylcytidine deamination frequencies as a function of A3A concentration.

To assess whether A3A deaminates 5MeC and C with similar efficiency, a customized oligodeoxynucleotide referred to as CER was designed to explore editing in matched dinucleotide contexts (Figure 2A). This substrate was incubated with highly active myc-His6-tagged A3A purified from HEK293 cells [18]. Purified A3G from baculovirus infected insect cells was used as negative control [1], [34]. DNA was recovered by 3DPCR. As can be seen from Figure 2B, DNA was recovered from the A3A-reaction down to a denaturation temperature of 77.3°C while the corresponding temperature for the A3G reaction was 82.2°C (Figure 2B). Interestingly, A3A and A3G enzymes purified from *E. coli* and baculovirus infected cells (not shown) gave similar editing frequency and context analysis. This precludes the necessity for any cellular partner in the deamination reaction. To generate site-specific editing frequencies DNA was cloned and sequenced from a number of reactions notably at 95°C and 84°C to avoid selection biases (Figure 2B). Using this assay, both matched 5MeC and C sites were edited efficiently, with the latter appearing slightly more susceptible (Figure 2C and 2D). However, as comparison of the ApC_6_ and Ap^m^C_10_ shows, occasionally 5MeC deamination could be as efficient as unmodified C (Figure 2D).

**Figure 3:**
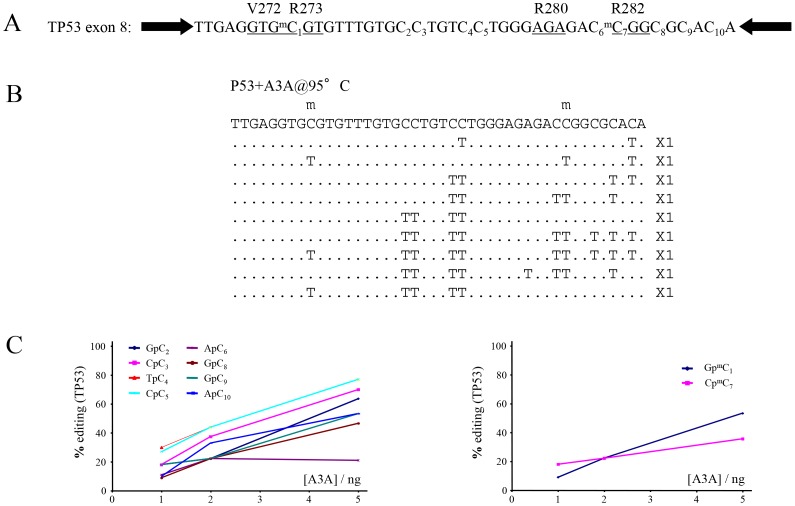
A3A deamination of an oligonuclotide harbouring two methylated CpGs that are TP53 mutation hotspots in cancers. A) The *TP53* target sequence is nested between two PCR primer targets (black arrows). The underlined triplets highlight the codons which are frequently substituted in cancers. B) A selection of deaminated *TP53* sequences with the number of mutant sequences shown to the right. C) Site specific cytidine and 5-methyl cytidine deamination frequencies as a function of A3A concentration.

As several *TP53* CpG methylation sites are mutational hotspots in cancer (www.iarc.fr/p53/), an oligodeoxynucleotide corresponding to part of exon 8 was synthesized with 5MeC incorporated at two known sites of methylation in codons 273 and 282 (Figure 3A). Following incubation with purified myc-His6-tagged A3A and recovery of products by standard PCR, both 5MeC and C were readily deaminated in a comparable manner (Figure 3B and 3C), in agreement with the observations for the CER oligonucleotide. In terms of pathology, it is plausible that many CG->TA mutations associated with cancer may be precipitated by PCD-catalyzed DNA cytidine deamination events hitherto attributed to spontaneous hydrolysis. Given that 5MeC is deaminated by A3A the singularity of 5MeCpG mutation hotspots in cancer probably has more to do with the relative efficiency of T:G mismatch repair compared to highly efficient U:G repair initiated by UNG [26]–[28].

These findings show that when over-expressed A3A is by far the most efficient human PCD at deaminating 5MeC DNA. They do not preclude some activity from other PCDs, notably AID, which is known to have relatively low catalytic activity [37], [38]. Although 3DPCR failed to pick evidence of hyperediting, it is known that 3DPCR underestimates lightly edited DNA molecules. That baculovirus derived A3G could occasionally edit a 5MeC residue in the CER oligodeoxynucleotide suggests that A3A is singular in terms of 5MeC deamination efficiency, rather than having 5MeC deamination as a unique property (Figure 2C).

**Figure 4:**
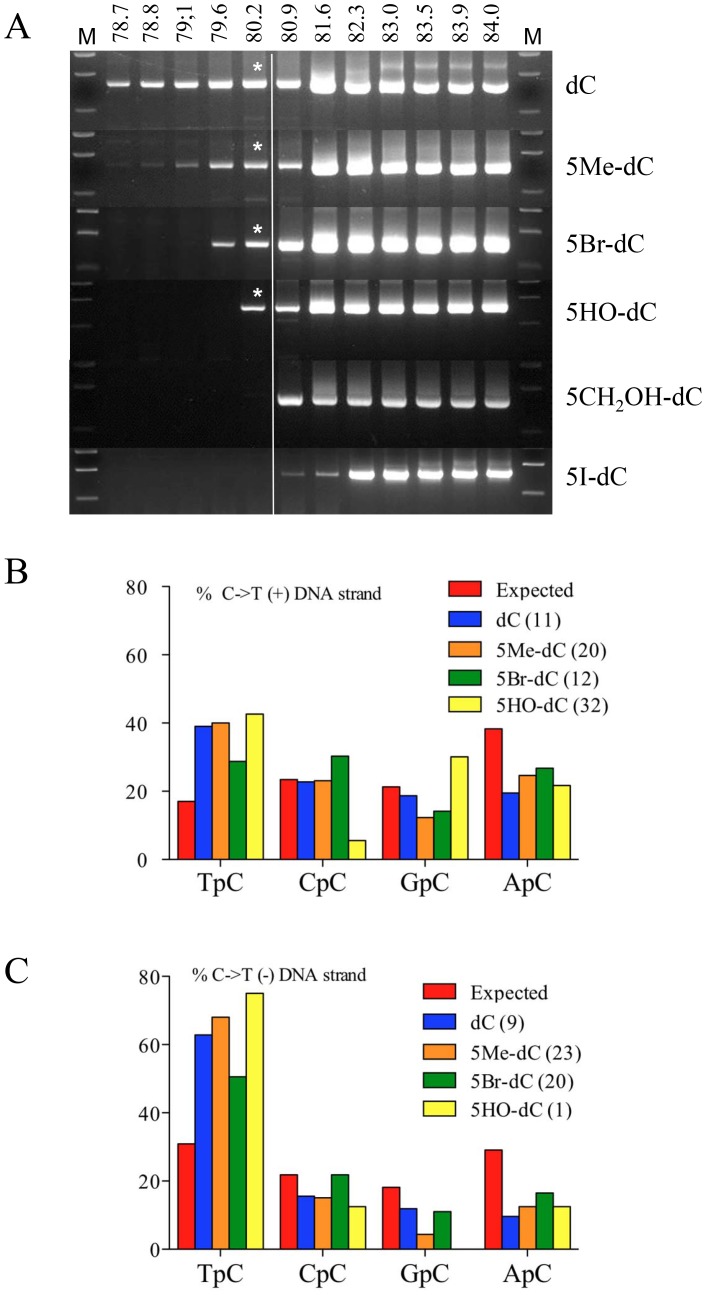
A3A can deaminate 5 bromo- and 5 hydroxy-cytidine ssDNA derivatives. A) 3DPCR analysis of recovered HIV DNA. Only for the 5Me, 5Br and 5HO derivatives were deaminated products recovered. Asterisks denote the 3DPCR products cloned and sequenced. B) 5′ Dinucleotide analysis of the deamination context. For both strands a clear preference for TpC was evident.

As A3A is able to deaminate 5MeC, we explored its capacity to deaminate other 5-modified cytidine residues in ssDNA. Totally substituted DNA products were made by PCR using 5-modified dCTP derivatives using Taq polymerase (Bioline, USA). We succeeded in making hydroxymethyl (5CH_2_OH-), hydroxy (5OH-), bromo (5Br-) and iodo (5I-) cytidine derivates. The same experimental procedure was adopted as described for Figure 1A. As can be seen in Figure 4A 3DPCR recovered DNA at or below the restrictive temperature of 80.2°C for the bromocytidine (79.6°C) and hydroxycytidine (80.2°C) derivatives, although compared to 5MeC, deamination was less efficient. Cloning and sequencing revealed that the bromo and hydroxycytidine adducts were indeed deaminated by A3A with deamination frequencies of between 36–57% and 2–26% per sequence. The dinucleotide context showed the invariant bias in favour of TpC, a trait for A3A (Figure 4B). The volumes of these 5-moeities are: hydroxy 19 Å^3^, methyl 31 Å^3^, bromo 33 Å^3^, iodo 38 Å^3^ and hydroxymethyl 41 Å^3^ [39]. As the iodo and hydroxymethyl derivatives were not deaminated it is possible that the A3A binding pocket cannot accommodate cytidine derivatives larger than 5-bromocytidine [39], [40].

As A3 editing is part of a DNA catabolic pathway [19], the problem of catabolizing modified DNA bases arises. 5MeC is found in nuclear and mitochondrial human DNA as well as bacterial DNA. Other recent reports have shown that A3A can deaminate 5MeC [41], [42]. However, that A3A can also edit free radical oxidation DNA products expands its catabolic role. As 5-chloro-dCTP is not commercially available, we were not able to explore the effect of A3A on a substrate akin to •OCl oxidized DNA. However, given that the volume of the 5-chloro moiety is 27 Å^3^, less than that of bromine (33 Å^3^) [39], it is likely that A3A could deaminate 5-chlorocytidine in ssDNA.

Once again A3A emerges as one of the most singular of human PCDs – it alone is able to hyperedit nuDNA with a mutation frequency approaching 0.5 as well as deaminating 5-substituted cytidine in ssDNA. Although hypermutation is synonymous with DNA catabolism, a little A3A editing might be compatible with cell survival. The repair of deaminated 5-modified cytidine residues proceeds by mechanisms that are not as efficient as for the G:U pair which invariably is initiated by the highly efficient enzyme UNG [26]–[28]. As 5MeCpG deamination hotspots characterize many genes associated with cancer [43], it is plausible that A3A is a major player in the onset of cancer.
